# Dental Calculus Arrest of Dental Caries

**DOI:** 10.13188/2377-987x.1000017

**Published:** 2016-02-12

**Authors:** Paul H. Keyes, Thomas E. Rams

**Affiliations:** 1 Formerly National Institute of Dental and Craniofacial Research, National Institutes of Health, Bethesda, Maryland, USA; presently retired, Washington, DC, USA; 2 Department of Periodontology and Oral Implantology, and Oral Microbiology Testing Service Laboratory, School of Dentistry, Department of Microbiology and Immunology, School of Medicine, Temple University, Philadelphia, Pennsylvania, USA

**Keywords:** Dental calculus, Dental caries, Dental plaque, Human, In vitro, Teeth

## Abstract

**Background:**

An inverse relationship between dental calculus mineralization and dental caries demineralization on teeth has been noted in some studies. Dental calculus may even form superficial layers over existing dental caries and arrest their progression, but this phenomenon has been only rarely documented and infrequently considered in the field of Cariology. To further assess the occurrence of dental calculus arrest of dental caries, this study evaluated a large number of extracted human teeth for the presence and location of dental caries, dental calculus, and dental plaque biofilms.

**Materials and methods:**

A total of 1,200 teeth were preserved in 10% buffered formal saline, and viewed while moist by a single experienced examiner using a research stereomicroscope at 15-25× magnification. Representative teeth were sectioned and photographed, and their dental plaque biofilms subjected to gram-stain examination with light microscopy at 100× magnification.

**Results:**

Dental calculus was observed on 1,140 (95%) of the extracted human teeth, and no dental carious lesions were found underlying dental calculus-covered surfaces on 1,139 of these teeth. However, dental calculus arrest of dental caries was found on one (0.54%) of 187 evaluated teeth that presented with unrestored proximal enamel caries. On the distal surface of a maxillary premolar tooth, dental calculus mineralization filled the outer surface cavitation of an incipient dental caries lesion. The dental calculus-covered carious lesion extended only slightly into enamel, and exhibited a brown pigmentation characteristic of inactive or arrested dental caries. In contrast, the tooth's mesial surface, without a superficial layer of dental calculus, had a large carious lesion going through enamel and deep into dentin.

**Conclusions:**

These observations further document the potential protective effects of dental calculus mineralization against dental caries.

## Introduction

Dental calculus deposition onto supragingival and subgingival tooth surfaces is traditionally viewed as detrimental to human oral health [1]. Supragingival calculus may contribute to development of gingival recession [2], and teeth positive for subgingival dental calculus experience a greater rate of clinical periodontal attachment loss in teenagers [3] and patients with untreated chronic or aggressive periodontitis [4,5]. However, the periodontopathic potential of dental calculus stems largely from unmineralized disease-associated bacterial biofilms coating its outer surfaces and nested within its structural lacunae and porosities [6,7], since dental calculus itself exhibits negligible pathogenicity when sterilized free of living microorganisms [8], and can even provide an adherent surface for junctional epithelium when disinfected [9].

Moreover, it is noteworthy that dental caries and dental calculus employ opposite biochemical processes in their oral cavity development. Dental caries, a multi-factorial infectious disease, triggers demineralization of susceptible teeth as a result of largely sucrose-driven acidogenic activity by mutans streptococci and other cariogenic bacterial species in dental plaque biofilms [10-12]. In contrast, dental calculus crystal formation relies upon progressive mineralization that occurs with precipitation of primarily calcium phosphate mineral salts onto tooth surfaces from salivary and gingival crevicular fluid secretions [13], even in the absence of dental plaque microorganisms [14].

As a result, it is not surprising that an inverse clinical relationship has been observed between dental calculus and dental caries. Leonard noted in 1926 that, “One seldom finds caries in the mouths of big eaters where much calculus is present, certainly never in the same areas with it” [15]. In six dentifrice clinical trials conducted between 1970-1986 and involving 6,284 children aged 11-13 years, dental caries prevalence at baseline, and its subsequent incidence over a three-year longitudinal period, was found on average to be 16-20% lower in children with supragingival dental calculus deposits, independent of fluoride exposure [16]. In another dentifrice clinical trial of 437 adults aged 20-65 years and selected for their propensity to form dental calculus, statistically significant negative correlation coeffcients were found on an individual subject basis between mean three-month dental calculus increment assessments and mean caries prevalence scores [17].

These findings suggest that fewer dental caries occur in persons with a high level of dental calculus formation, whereas a greater dental caries risk is associated with no or little dental calculus mineralization. Other studies have failed to find a significant relationship between dental calculus and dental caries [16,17], but their conclusions may have been affected by small sample sizes, and inadequate accounting for potential confounding factors like poor oral hygiene and older-aged study subjects [16,17].

Dental calculus may even form superficial layers over existing dental caries lesions and arrest their progression, but this phenomenon has been only rarely documented and infrequently considered in the field of Cariology [18]. Early observations reported clinical arrest of untreated dental caries by dental calculus deposits developing in diabetic children as they were brought into glycemic control with a low carbohydrate diet rich in mineral salts [19]. In hamster animal studies, dental calculus was seen forming over the outer surface of root caries lesions and arresting their progression when high-sucrose diets favoring demineralization and dental caries were changed to a protein-based laboratory chow favoring mineralization and dental calculus development [20,21].

Few data are available on the frequency with which dental calculus may be involved in arresting existing dental caries. In a study of 2,300 extracted human posterior teeth, a total of 14 teeth, for a prevalence rate of 0.56%, exhibited a superficial layer of dental calculus precipitated over the outer surface of proximal dental caries exhibiting brown pigmentation associated with arrested lesions [22]. To further document the occurrence of dental calculus arrest of dental caries, this study assessed 1,200 extracted human teeth for presence and location of dental caries, dental calculus, and dental plaque biofilms.

## Materials and Methods

A total of 1,200 extracted non-deciduous human teeth (277 incisors, 138 canines, 415 premolars, and 370 molars) were obtained over a period of several years in the mid-1960s from the United States Public Health Service dental clinic at the National Institutes of Health, Bethesda, Maryland, USA, without any accompanying patient demographic information, dental diagnosis, dental or medical history data, or reason for extraction. The teeth were preserved in 10% buffered formal saline, and viewed while moist by a single experienced examiner (author P.H. Keyes) using a research stereomicroscope at 15-25× magnification for the presence and location of dental caries, dental calculus, and dental plaque biofilms. Representative teeth were sectioned and photographed, and their dental plaque biofilms subjected to gram-stain examination with light microscopy at 100× magnification.

Approval for this study was provided by the National Institutes of Health, and, for the present data analysis, by the Temple University Human Subjects Protections Institutional Review Board.

## Results

Mineralized deposits of dental calculus, ranging from slight to heavy, were observed on 1,140 (95%) of the evaluated teeth. No dental carious lesions were found underlying dental calculus-covered surfaces on 1,139 of the extracted human teeth.

**Figure 1:**
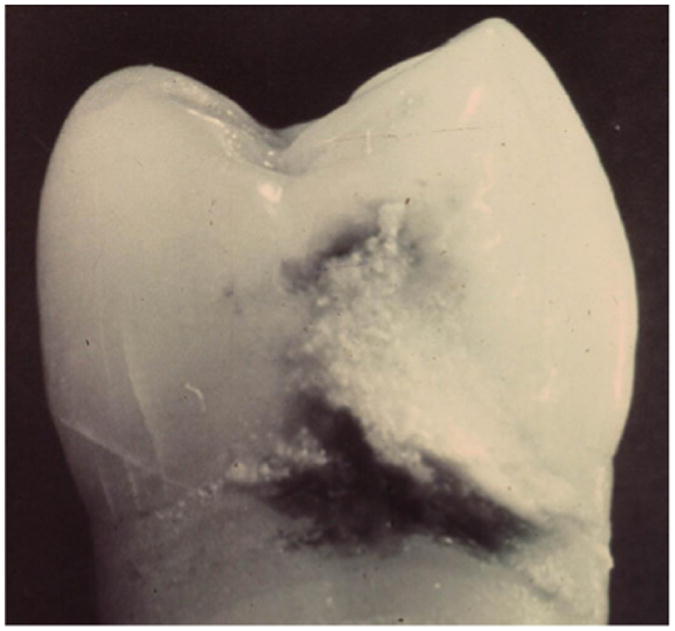
Dental calculus covering dental caries Dental calculus deposition on distal surface of a maxillary premolar which extends from cementoenamel junction to the outer surface of incipient enamel dental caries near contact area.

**Figure 2:**
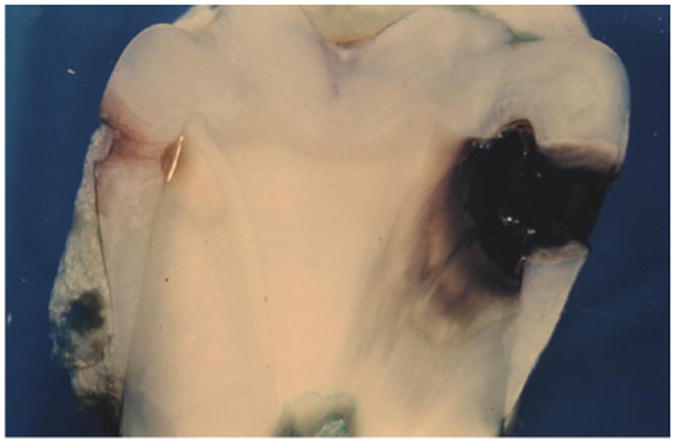
Dental caries progression with and without overlying dental calculus deposits Cross-section of a maxillary premolar shows incipient enamel dental caries covered with superficial layer of dental calculus on distal surface (left) (white line at junction of enamel and dentin is processing artifact), and large dental carious lesion on mesial surface extending deep into dentin without overlying dental calculus (right).

**Figure 3:**
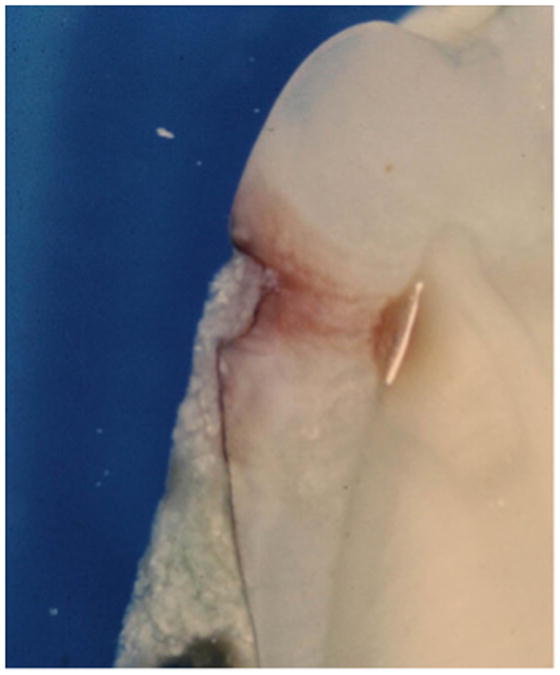
Dental calculus deposition into dental carious lesion Enlarged view of distal tooth surface with dental calculus “filling” in incipient enamel dental carious lesion (white line at junction of enamel and dentin is processing artifact).

However, one maxillary premolar had on its distal surface a superficial layer of dental calculus overlying and filling the outer surface cavitation of an incipient proximal dental caries lesion (Figure 1). The dental caries lesion was found in cross-section to extend only slightly into enamel, with minimal changes to underlying dentin, and exhibited brown pigmentation characteristic of inactive or arrested dental caries (Figures 2 and 3). In contrast, the tooth's mesial surface, without a superficial layer of dental calculus on its outer surface, had a large carious lesion going through enamel and deep into dentin (Figure 2), with dark pigmentation of demineralized dentin and underlying dentinal tubules. The single tooth revealing a layer of dental calculus over an arrested proximal carious lesion represented 0.54% of 187 evaluated teeth that presented with unrestored proximal enamel caries. No information was available about the patient from whom this tooth was extracted.

**Figure 4:**
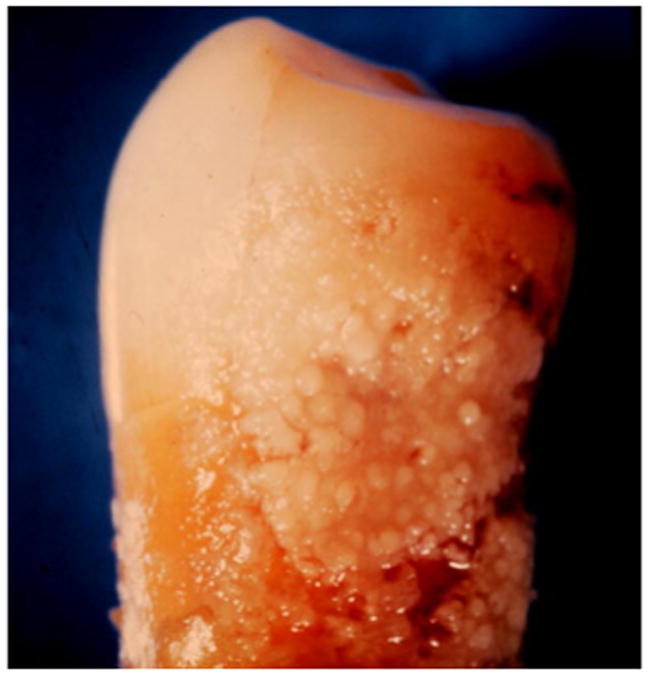
Dental plaque microbial biofilm Example of thick and highly adherent dental plaque biofilm established on proximal surface of a premolar tooth.

In addition to these findings, 360 (30%) of the 1,200 extracted teeth studied had proximal enamel surface caries, of which 173 (48.1%) were treated with dental restorations. Root caries lesions were noted on 432 (36%) teeth. A total of 804 (67%) extracted teeth had facial tooth surfaces clean, highly-polished, and devoid of dental plaque biofilms, indicative of good patient home plaque control at these tooth sites. Less accessible proximal areas and root surfaces on 1,056 (88%) teeth revealed adherent gummy growth characteristic of dental plaque biofilms and flawed patient home plaque control (Figure 4). Microscopic examination of gram-stained smears of a subset of these gummy growths revealed almost exclusively bacterial cells.

## Discussion

These study observations suggest a protective effect of dental calculus deposition against dental caries. No dental carious lesions were found beneath dental calculus-covered surfaces on 1,139 extracted human teeth, and a dental calculus “filling” was found overlying the outer surface of an incipient proximal dental caries lesion on a human maxillary premolar. This latter observation of dental calculus precipitating over existing dental caries occurred on 0.54% of 187 teeth evaluated with unrestored proximal enamel caries, which is nearly identical to the 0.56% prevalence reported for this phenomenon in a previous study of extracted human teeth [22].

The temporal sequence as to when dental calculus and dental caries developed on this particular tooth with a dental calculus “filling” is not known. However, it is unlikely that dental calculus and dental caries developed simultaneously on the same proximal tooth surface location. Tanzer et al. successfully induced dental calculus and dental caries at the same time in rats, but only in different parts of the dentition, and not on the same tooth surface [23]. Similarly, on dentin slabs worn in removable appliances by human volunteers, experimental dentin demineralization was noted only in areas surrounding dental calculus deposits, and not underneath them [24].

In this regard, Driessens et al. pointed out that since the solubility of dental calculus is much higher than enamel, it is not possible for enamel demineralization to occur beneath mineralized dental calculus deposits on tooth surfaces [22]. In other words, due to its greater solubility in acidic environments, dental calculus on a tooth surface would have a greater potential to demineralize prior to demineralization of any underlying enamel. The greater acidic demineralization susceptibility of dental calculus, as compared to enamel, precludes formation of dental caries underneath established dental calculus deposits [22]. Based on this, it is postulated for the maxillary premolar in the present study (Figures 1-3), that the observed proximal dental caries developed prior to deposition of dental calculus on the distal tooth surface. As a result, it is postulated that the dental calculus deposits overlying the dental caries on the distal tooth surface formed secondarily after an unknown change occurred in the patient's oral cavity which then favored mineralization at that point, rather than continued demineralization. Factors triggering a change in the patient's oral cavity mineralization versus demineralization potential could not be determined in the present study.

However, this proposed scenario is consistent with observations in hamster animal model studies, where dietary changes from one high in sucrose, to one rich in protein, altered oral mineralization conditions and promoted dental calculus precipitation onto existing dental carious lesions [20,21]. Similarly, a sucrose-rich, phosphate-poor diet in rats was shown to induce high dental caries activity and little dental calculus formation, whereas a diet poor in sucrose and rich in phosphate increased dental calculus formation and lessened dental caries [25].

Interestingly, humans going onto a low-carbohydrate, high-protein diet are also reported to experience a marked increase in dental calculus deposition [26]. This may be due to the combined effects of a diminished consumption of fermentable carbohydrates reducing acidogenic activity of oral cariogenic microorganisms [27], and high protein intake increasing salivary urea secretions, which, after being metabolized into ammonia and CO_2_ by oral ureolytic bacteria, help increase dental plaque pH to more alkaline levels and favor its calcification into dental calculus [28].

Also consistent with the concept that dental calculus and dental caries are jointly incompatible, the major cariogenic bacterial species, *Streptococcus mutans*, is not isolated from the cultivable microbiota adherent to mature supragingival or subgingival dental calculus deposits [29].

These findings support the views of Mühlemann [30] on dental calculus, who commented that “it is difficult to consider the calcifying processes as primarily pathogenic”, and that “the transformation of metabolically active microbial colonies into non-living calcified masses could be interpreted as a protective mechanism”. It may be that dental calculus mineralization as a protective host response does not precipitate rapidly enough onto tooth surfaces to adequately fossilize pathogenic dental plaque biofilms and prevent their ability to induce dental caries and periodontal diseases. Therapeutic efforts may be better directed at controlling pathogenic bacterial biofilms which may colonize dental calculus, as compared to a primary focus on stopping dental calculus mineralization onto tooth surfaces. As pointed out by Keyes and Shern [31], it is easier and prognostically more favorable to periodically remove dental calculus calcifications from a given tooth surface than to treat root caries lesions on it. Further research is needed to better define the protective versus pathogenic potentials of dental calculus in the oral cavity.

## Conclusion

In conclusion, this study of extracted human teeth found dental calculus depositing over the outer surface of an incipient proximal dental caries lesion on the distal surface of one maxillary premolar, and arresting its progression, for a prevalence of 0.54% among evaluated teeth with unrestored proximal enamel caries. No dental carious lesions were found underlying dental calculus-covered surfaces on 1,139 other extracted human teeth. These observations further document the potential protective effects of dental calculus mineralization against dental caries.
